# Adaptive response triggered by the repeated SCUBA diving is reflected in cardiovascular, muscular, and immune biomarkers

**DOI:** 10.14814/phy2.14691

**Published:** 2021-01-19

**Authors:** Marko Žarak, Antonija Perović, Marina Njire Bratičević, Sandra Šupraha Goreta, Jerka Dumić

**Affiliations:** ^1^ Clinical Department of Laboratory Diagnostics Dubrava University Hospital Zagreb Croatia; ^2^ Department of Laboratory Diagnostics Dubrovnik General Hospital Dubrovnik Croatia; ^3^ Faculty of Pharmacy and Biochemistry Department of Biochemistry and Molecular Biology University of Zagreb Zagreb Croatia

**Keywords:** endothelin‐1, galectin‐3, IL‐6, myoglobin, NT‐proBNP, SCUBA diving

## Abstract

It has been shown that one recreational SCUBA (rSCUBA) diving session is sufficient to cause changes in plasma level of cardiovascular (CV) and muscular biomarkers. To explore whether repetitive rSCUBA diving triggers an adaptive response of the CV, muscular, and immune system, we measured the cardiac damage (NT‐proBNP, hs‐TnI, and CK‐MB), muscle damage (myoglobin (Mb), galectin‐3, CK, and LDH), vascular endothelial activation (ET‐1 and VEGF), and inflammatory (leukocyte count (Lkc), CRP, and IL‐6) biomarkers. A longitudinal intervention study included divers (N = 14) who conducted one dive per week over 5 weeks at the depth of 20–30 m for 30 min after a non‐dive period of 5 months. The blood samples were collected before and after the first, third, and fifth dives and specific biomarkers were measured in plasma or serum by the standard laboratory methods. The concentrations of the majority of measured biomarkers increased after every single dive; the exception was ET‐1 concentration that decreased. The cumulative effect of five dives has been reflected in diminishing changes in hs‐TnI, Mb, galectin‐3, ET‐1, VEGF, and IL‐6 levels, and more pronounced increases in NT‐proBNP and hs‐CRP levels. The median values of all measured biomarkers in all time points, except Mb, remained within the corresponding reference range. Repeatedly performed rSCUBA diving activates an adaptive response of the CV, muscular, and immune system that is reflected in changes in the specific biomarker concentration.

## Graphical abstract

Under challenging environmental conditions, stress forces organisms to adapt to the rapidly changing surroundings. This is the first study on the cumulative effect of rSCUBA diving on the blood concentration of the majority of the selected biomarkers. Repeatedly performed recreational SCUBA diving triggered activation of an adaptive response of the cardiovascular, muscular, and immune systems that were reflected in changes in the specific biomarkers. No clinically relevant impairment of any observed systems was seen, but all measured biomarkers showed different dynamics of change with repeatedly performed rSCUBA diving. Studies on the cumulative effect of recreational SCUBA diving should be carefully designed, because the frequency of repeated dives seems to be crucial for triggering the adaptive response that could be recorded by the blood levels of the specific biomarkers.

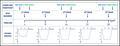

## 1 INTRODUCTION

Positive effects of regular, moderate‐intensity exercise on physical fitness and overall health of humans are well known. Active life and practicing sports have been confirmed to improve cardiovascular (CV) health (Golbidi & Laher, 2012; Joyner & Green, 2009; Lavie et al., 2015), and reduce morbidity and mortality in the general population (Kruk, 2007). One of the proposed mechanisms by which exercise contributes to health benefits is a reduction of basal inflammation (Beavers et al., 2010; Hamer et al., 2012; Pedersen, 2017). However, many extreme sports, including SCUBA (self‐contained underwater breathing apparatus) diving, that have an increasing number of new recreational athletes every year, have not been intensively investigated in regard to their effects on the human body (Åsmul et al., 2017), so the health implications as well as molecular (patho)physiology underlying each of them remain to be clarified.

According to the Universal standards and procedures of the World Underwater Federation, recreational or sports SCUBA diving is a form of diving up to the depth of 40 m with direct access to the surface (without decompression stop) using compressed air or nitrox (a mixture of oxygen and nitrogen where the oxygen content does not exceed 40%) as a breathing gas (CMAS, 2012). As a specific form of physical activity, due to challenging environmental conditions (immersion, coldness, hyperoxia, and high external pressure), it can cause particular stress in the human body. In order to maintain homeostasis, the body activates numerous molecular mechanisms that lead to the functional adaptation of different organs and tissues, such as heart (Breskovic et al., 2011), lung (Skogstad et al., 2000), muscles (Žarak et al., 2020), and blood vessels (Marinovic et al., 2012), and activation of the immune system (Sureda et al., 2014). Our previous study on the effect of a single session of recreational SCUBA (rSCUBA) diving on the CV system (Žarak et al., 2020) showed moderate but reversible changes in biomarkers of cardiac (N‐terminal prohormone of brain natriuretic peptide (NT‐proBNP) and high‐sensitivity troponin I (hs‐TnI)) and muscle damage (myoglobin (Mb) and galectin‐3 (Gal‐3)), and vascular endothelial activation (vascular endothelial growth factor (VEGF) and endothelin‐1 (ET‐1)). Other studies found similar results for NT‐proBNP after both single rSCUBA diving sessions (Gempp et al., 2005; Grassi et al., 2009; Passino et al., 2011) and technical (dives deeper than 40 m with an obligatory decompression stop (CMAS, 2012)) diving sessions (Ljubkovic et al., 2010; Marinovic et al., 2010). Further, Sureda et al. (2012) found similar results; a decrease in ET‐1 and an increase in VEGF in a group of divers who performed a single technical SCUBA diving session. Nevertheless, there are only few studies on the effects of the repeated SCUBA diving; in two of them, NT‐proBNP concentration was measured after repeated technical SCUBA diving (Ljubkovic et al., 2010; Marinovic et al., 2010), whereas in the third study, ET‐1 concentration was monitored after repeated rSCUBA diving (Bilopavlovic et al., 2013), but no cumulative effect has been observed. In general, information on the cumulative effect of the repeated SCUBA diving, either recreational or technical, is scarce.

Therefore, to contribute to the elucidation of the cumulative effect of the repetitive rSCUBA diving on the CV, muscular, and immune systems, we examined the effects of five consecutive rSCUBA dives (one per week) after 5 months non‐dive period by measuring plasma or serum concentration of cardiac (hs‐TnI, NT‐proBNP, creatine kinase myocardial band (CK‐MB), Gal‐3, and high‐sensitivity CRP (hs‐CRP)) and muscle damage (Mb, Gal‐3, creatine kinase (CK), and lactate dehydrogenase (LDH)), vascular endothelial activation (ET‐1 and VEGF), and inflammatory (leukocyte count (Lkc), C‐reactive protein (CRP), and interleukin‐6 (IL‐6)) biomarkers.

## 2 MATERIALS AND METHODS

### 2.1 Subjects

This longitudinal intervention study included 14 male recreational divers, median age (range) 42 (19–54) years with diving experience of between 3 and 20 years and the number of dives per year less than 30. Divers were not professional athletes and had not practiced SCUBA diving for at least 5 months (during the winter period). All divers had a valid medical certificate for diving, and none of them had symptoms of any acute or chronic disease, excluded by taking detailed anamnestic data before the study and by laboratory testing. Before giving their written consent to participate, the subjects were informed on the purpose and demands of the study. The study was designed according to the Declaration of Helsinki and was approved by the Ethical Committee of the University of Zagreb Faculty of Pharmacy and Biochemistry.

### 2.2 Study design and blood collection

**FIGURE 1:**
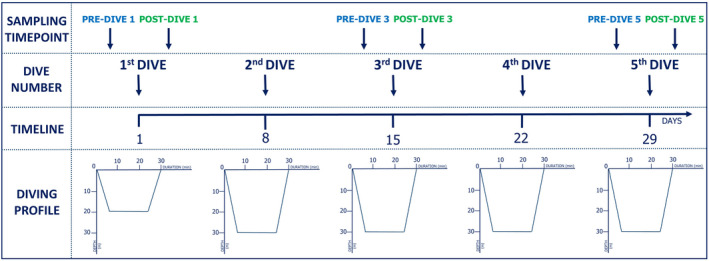
Study design and blood collection time points

Experimental dives were conducted at the Adriatic seaside from March to April 2019. All divers performed five consecutive dives (one dive per week); the first dive at the maximum depth of 20 m for a total time of 30 min, and other four dives at the maximum depth of 30 m for a total time of 30 min (Figure 1). All five dives were carried out at the same time in the morning, with gradual immersion to the maximum depth (with the descending velocity of 10 m/min) and a gradual return to the surface without a decompression stop (ascending velocity of 9 m/min). Diving equipment consisted of wetsuits, dive computers, and open‐circuit scuba diving apparatus with compressed air. The sea temperature was between 12 and 15 °C and the air temperature was between 16 and 20 °C.

Six blood samples were taken from all the subjects for the time‐course evaluation: before (pre‐dive) and immediately after (post‐dive) on the first, third, and fifth dives (Figure 1). Blood samples were drawn from the antecubital vein of subjects, directly into vacuum tubes with K_2_EDTA and clot activator. Blood samples were centrifuged within 1 h after blood sampling and collected plasma and serum samples were stored at −80°C.

### 2.3 Methods

NT‐proBNP plasma concentration was determined by chemiluminescence test on Abbott Architect i1000SR (Abbott Diagnostics), while hs‐TnI and IL‐6 plasma concentrations were determined by chemiluminescence assays on Beckman Coulter UniCel DxI 600 (Beckman Coulter). Serum concentration of CRP and plasma concentration of hs‐CRP were measured by CRP Latex (immunoturbidimetry) on a Beckman Coulter AU 680, using the corresponding calibrators. Myoglobin was measured in plasma by Myoglobin (immunoturbidimetry) test on the same analyzer as well as the serum CK, CK‐MB, and LDH catalytic concentrations. Lkc was determined by Abbott Cell Dyn Ruby hematology analyzer in whole blood samples within 1 h after sampling. Plasma concentrations of Gal‐3, ET‐1, and VEGF were measured by immunochemical ELISA using the Quantikine ELISA Human assays (R&D Systems Inc.) on the automated ELISA Siemens BEP 2000 Advance (Siemens Healthcare Diagnostics Walpole). All the procedures, including all pre‐analytics, analytics, post‐analytics, and quality control, have been following the standard for the accreditation of medical laboratories EN ISO 15189.

### 2.4 Statistical analysis

According to the data distribution (tested using Shapiro‐Wilk test), only non‐parametric tests were used, and the data were presented as the median and interquartile range (IQR). The differences between pre‐ and post‐dive results were tested using the Wilcoxon signed‐rank test, while differences between all pre‐dive results as well as all post‐dive results were assessed using the Friedman ANOVA test. When Friedman's test was positive (*p* < 0.05), Dunn's pairwise post‐hoc test was performed to determine which variable was significantly different from the other variables. *p*‐values obtained by the Dunn's test were adjusted by a Bonferroni correction; *p*‐values are automatically multiplied by the number of tests (N = 3) being carried out. Statistical analysis was performed using SPSS computer program (IBM SPSS Statistics 27.0, SPSS Inc.). The level of significance was set at *p* < 0.05.

The sample size was estimated by the power test analysis through the level of the effects (changes wanted to be observed) taken from previous studies (Ferrer et al., 2007; Hättasch et al., 2014; Kim et al., 2015; Toft et al., 2011) for the main outcome variables (NT‐proBNP, hs‐TnI, Gal‐3, IL‐6, LDH, and CK). The estimated minimum number of samples (with the minimal power of the study 90% (*β* = 0.10) and 5% significance to detect a difference (*α* = 0.05)) is in agreement with the number of subjects involved in the study (N = 14).

## 3 RESULTS

To investigate the effects of repetitive rSCUBA diving (five dives, one per week) on the cardiac, muscular, vascular, and inflammatory biomarkers, we conducted a longitudinal intervention study and measured their plasma or serum concentrations in six time points: before and immediately after the first, third, and fifth dives, whereas after the second and fourth dives, samples have not been collected.

The anthropometric data were expressed as median and IQR; diver's height 1.80 (1.76–1.86) m, weight 85 (77–93) kg, and body mass index (BMI) 26.4 (23.5–28.5) kg/m^2^, respectively. The median values of all measured biomarkers in all time points remained within the corresponding reference range (except for Mb, which was slightly increased after the first and third dives), suggesting that there were no clinically relevant changes in concentrations of measured biomarkers that suggest that no pathological events have been found.

### 3.1 Inflammatory biomarkers (Lkc, CRP, and IL‐6)

**FIGURE 2:**
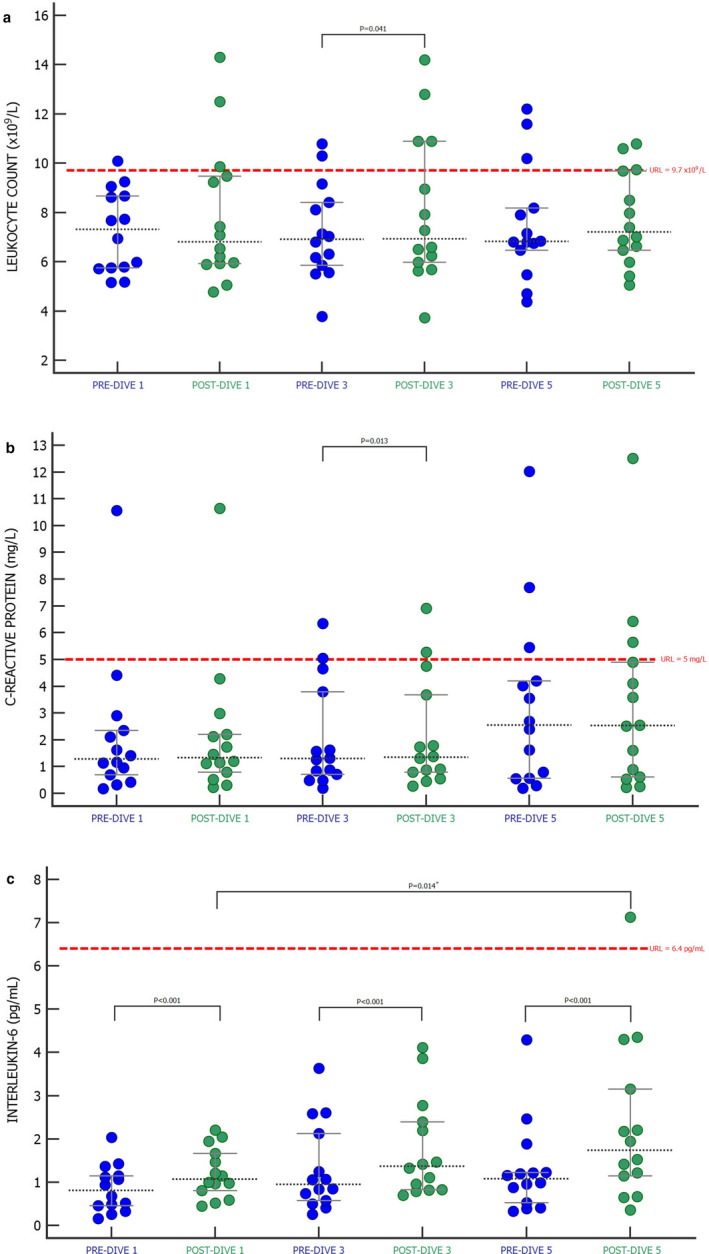
The effect of SCUBA diving on leukocyte count (a), C‐reactive protein (b), and interleukin‐6 (c) presented as Dots plot (all data) with corresponding median value and IQR ranges. PRE‐DIVE 1, 3, and 5—measuring points immediately before the first, third, and fifth dives. POST‐DIVE 1, 3, and 5—measuring points immediately after the first, third, and fifth dives. Differences between pre‐ and corresponding post‐dive values of every performed dive were tested with Wilcoxon singed‐rank test (*p* < 0.05), while differences between all pre‐ and all post‐dive values were tested with Friedman ANOVA test and Dunn's post‐hoc test. *Significance values have been adjusted by the Bonferroni correction for multiple tests. URL—upper reference level

CRP and IL‐6 concentrations as well as Lkc have been traditionally used to measure the extent of inflammation, so these markers have been analyzed to investigate the dynamics of change with rSCUBA diving (Figure 2). No increase above the upper reference limit (URL) of any of these biomarkers has been observed. Lkc (Figure 2a) and CRP concentrations (Figure 2b) did not show statistically significant changes when the post‐dives 1 and 5 were compared with the corresponding pre‐dive values, and only slight increases in both biomarkers were observed after the third dive (Lkc *p* = 0.041; CRP *p* = 0.013). When only pre‐dive and only post‐dive values of Lkc and CRP concentrations were compared, no statistically significant changes were found. However, a statistically significant increase in IL‐6 concentration was observed after diving at all time points (*p* < 0.001), and these increases grew after every preformed dive (Figure 2c). When comparing only pre‐dive or only post‐dive IL‐6 values, significantly higher post‐dive 5 vs. post‐dive 1 (*p* = 0.014) value was observed (Figure 2c).

### 3.2 Cardiac damage and stretch biomarkers (CK‐MB, hs‐TnI, NT‐proBNP, and hs‐CRP)

**FIGURE 3:**
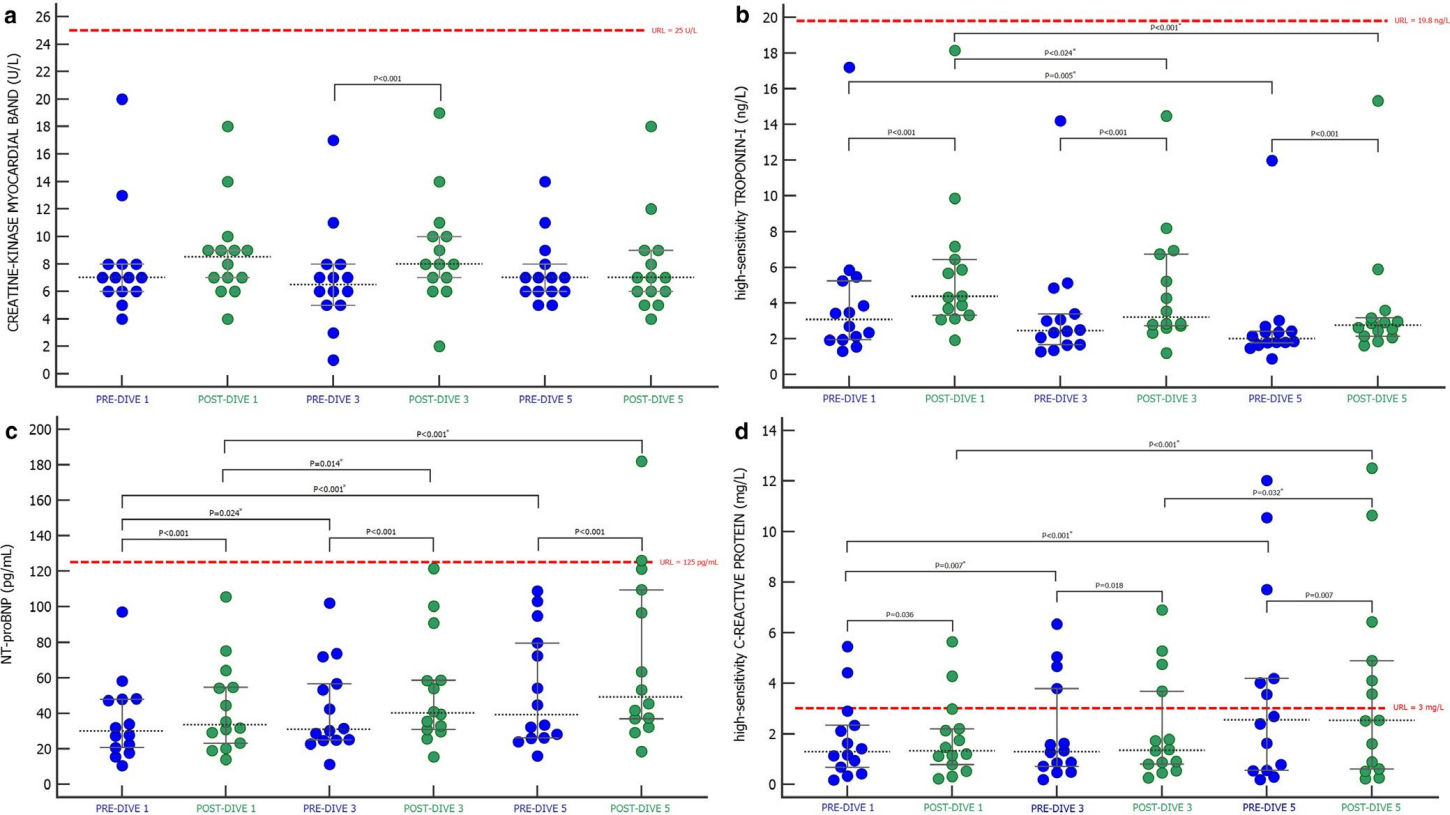
The effect of SCUBA diving on creatine kinase myocardial band (a), high‐sensitivity troponin I (b), N‐ terminal prohormone of brain natriuretic peptide (c), and high‐sensitivity C‐reactive protein (d) presented as Dots plot (all data) with corresponding median value and IQR ranges. PRE‐DIVE 1, 3, and 5—measuring points immediately before the first, third, and fifth dives. POST‐DIVE 1, 3, and 5—measuring points immediately after the first, third, and fifth dives. Differences between pre‐ and corresponding post‐dive values of every performed dive were tested with Wilcoxon singed‐rank test (*p* < 0.05), while differences between all pre‐ and all post‐dive values were tested with Friedman ANOVA test and Dunn's post‐hoc test. *Significance values have been adjusted by the Bonferroni correction for multiple tests. URL—upper reference level

To examine the effects of repeated rSCUBA on cardiomyocytes, the cardiac damage biomarkers, CK‐MB, hs‐TnI, and hs‐CRP, and the biomarker of stress and stretch within the myocardial walls, NT‐proBNP, were measured. Although all remained within the corresponding reference range, the changes in the concentrations of measured biomarkers showed different dynamics (Figure 3). CK‐MB catalytic concentration (Figure 3a) did not significantly change after first and fifth dives as compared to the corresponding pre‐dive values, whereas after third dive, it was slightly increased as compared to the pre‐dive value (*p* < 0.001). The comparison of only pre‐dive and only post‐dive values revealed no statistically significant changes in CK‐MB values. All post‐dive values of hs‐TnI and NT‐proBNP were significantly higher than the corresponding pre‐dive values (*p* < 0.001) (Figure 3b,c). Statistically significant increases in hs‐CRP concentration after first (*p* = 0.036), third (*p* = 0.018), and fifth (*p* = 0.007) dives were detected (Figure 3d). When only corresponding pre‐dive and only corresponding post‐dive values of hs‐TnI, NT‐proBNP, and hs‐CRP were compared, statistically significant decreases of pre‐ and post‐dive values of hs‐TnI (Figure 3b) and statistically significant increases of pre‐ and post‐dive values of NT‐proBNP (Figure 3c) and hs‐CRP (Figure 3d) were observed. When the hs‐TnI pre‐dive and post‐dive values were compared, the pre‐dive 1 value was statistically lower than the pre‐dive 5 value (*p* = 0.005), as well as the post‐dive 1 as compared to the post‐dive 3 and 5 values (*p* = 0.024 and *p* < 0.001, respectively). NT‐proBNP and hs‐CRP pre‐dive 1 concentrations were significantly higher as compared to the pre‐dive 5 values (*p* < 0.001 and *p* = 0.007, respectively), as well as the post‐dive 3 as compared to the post‐dive 5 values (both *p* < 0.001). NT‐proBNP post‐dive 1 concentration was significantly higher than the post‐dive 3 value (*p* = 0.014). hs‐CRP post‐dive 3 value significantly increased as compared to the post‐dive 5 value (*p* = 0.032).

### 3.3 Biomarkers of myocyte membrane integrity (CK, LDH, Mb, and Gal‐3)

**FIGURE 4:**
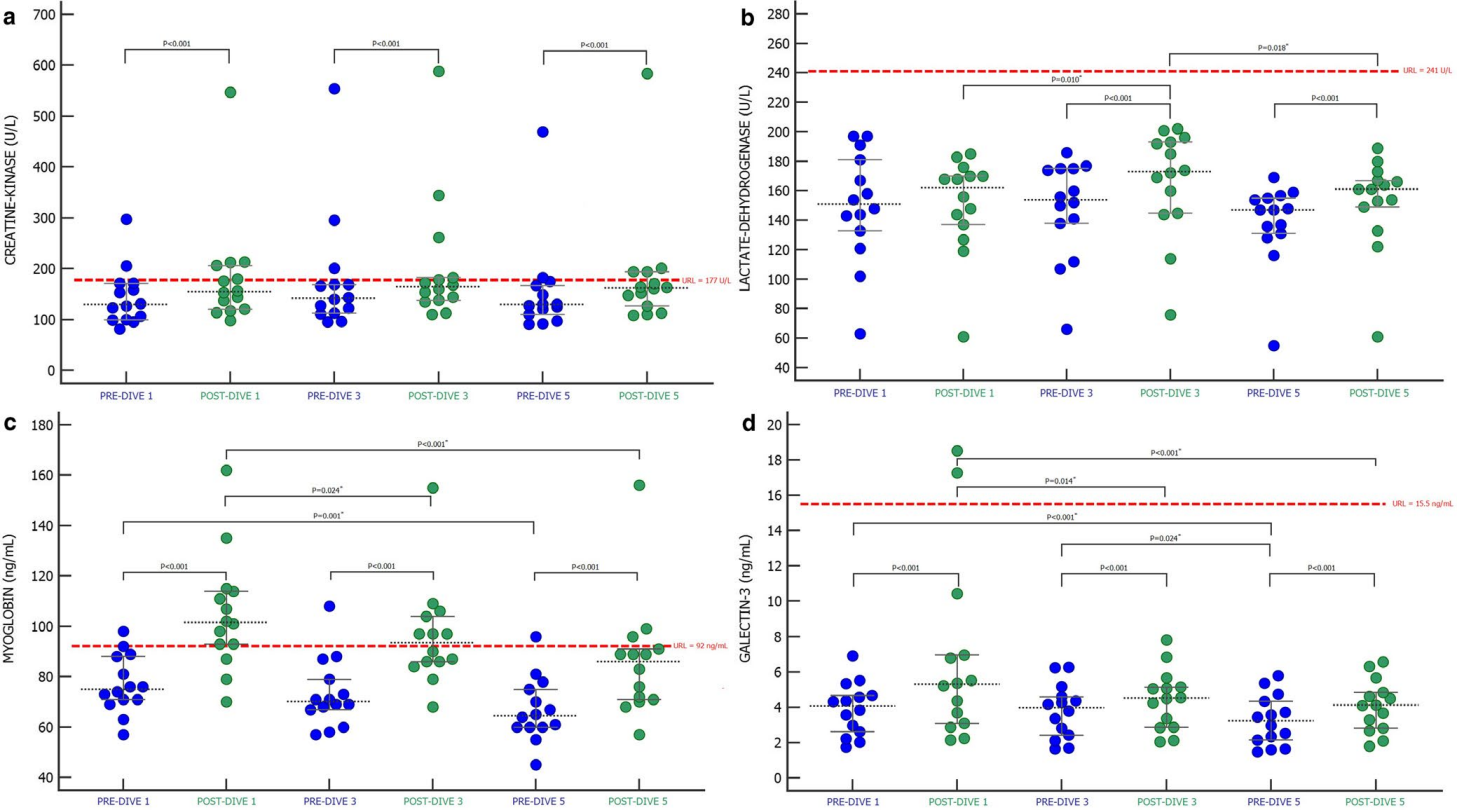
The effect of SCUBA diving on creatine kinase (a), lactate dehydrogenase (b), myoglobin (c), and galectin‐3 (d) presented as Dots plot (all data) with corresponding median value and IQR ranges. PRE‐DIVE 1, 3, and 5—measuring points immediately before the first, third, and fifth dives. POST‐DIVE 1, 3, and 5—measuring points immediately after the first, third, and fifth dives. Differences between pre‐ and corresponding post‐dive values of every performed dive were tested with Wilcoxon singed‐rank test (*p* < 0.05), while differences between all pre‐ and all post‐dive values were tested with Friedman ANOVA test and Dunn's post‐hoc test. *Significance values have been adjusted by the Bonferroni correction for multiple tests. URL—upper reference level

To explore the effects of repeated rSCUBA diving on muscles (myocyte membrane integrity) the catalytic concentration of CK and LDH as well as concentration of Mb and Gal‐3 were measured (Figure 4). CK catalytic concentration increased after diving in all time points (*p* < 0.001), but neither changes in pre‐dive nor post‐dive values were observed (Figure 4a). LDH catalytic concentration also increased after diving at all time points, but only after third and fifth dives increases were statistically significant (*p* < 0.001) (Figure 4b). When LDH post‐dive values were compared, the post‐dive 3 value was significantly higher than the post‐dive 1 value (*p* = 0.010) and the post‐dive 5 value lower than the post‐dive 3 value (*p* = 0.018). Increases in post‐dive plasma concentrations of both Mb and Gal‐3 at all time points were observed (Mb *p* < 0.001, Figure 4c; Gal‐3 *p* < 0.001, Figure 4d). Yet, both, pre‐dive and post‐dive values of both biomarkers were getting significantly lower after every performed dive (Figure 4c,d).

### 3.4 Biomarkers of vascular endothelium activity (VEGF and ET‐1)

**FIGURE 5:**
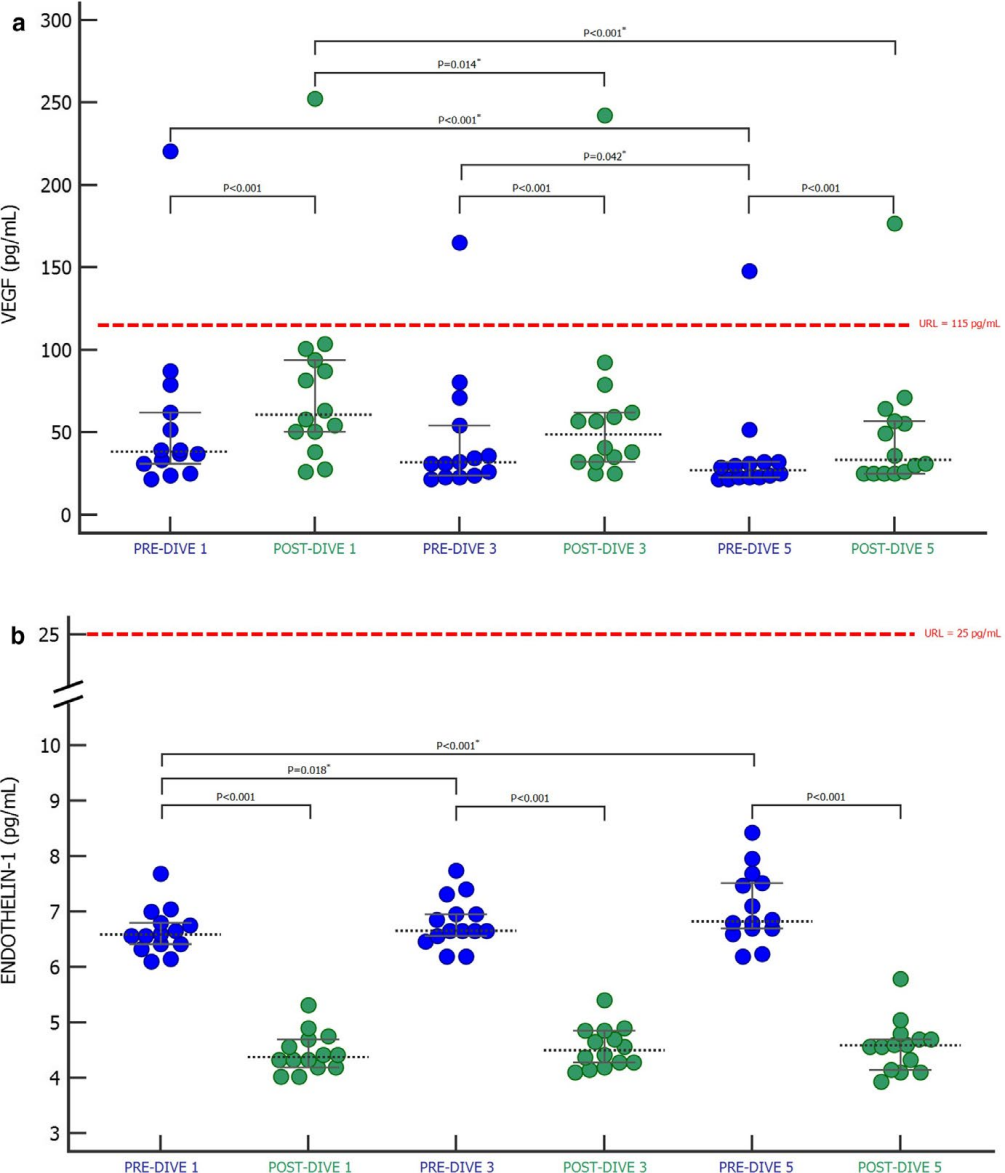
The effect of SCUBA diving on vascular endothelial growth factor (a) and endothelin‐1 (b) presented as Dots plot (all data) with corresponding median value and IQR ranges. PRE‐DIVE 1, 3, and 5—measuring points immediately before the first, third, and fifth dives. POST‐DIVE 1, 3, and 5—measuring points immediately after the first, third, and fifth dives. Differences between pre‐ and corresponding post‐dive values of every performed dive were tested with Wilcoxon singed‐rank test (*p* < 0.05), while differences between all pre‐ and all post‐dive values were tested with Friedman ANOVA test and Dunn's post‐hoc test. *Significance values have been adjusted by the Bonferroni correction for multiple tests. URL—upper reference level

The effect of repeated rSCUBA diving on the activity of vascular endothelium was estimated by the measurement of VEGF and ET‐1 concentrations (Figure 5). After every performed dive, a statistically significant increase in VEGF concentration (*p* < 0.001; Figure 5a) and a decrease in ET‐1 concentration (*p* < 0.001; Figure 5b) were noted. A comparison of only pre‐dive and only post‐dive values of VEGF showed a statistically significant continuous decrease in both value groups (Figure 5a; pre‐dive values *p* < 0.001; post‐dive values *p* < 0.001). Yet, the pre‐dive values of ET‐1 continuously increased (*p* < 0.001), but post‐dive values have remained unchanged (*p* = 0.411) (Figure 5b). Consequently, decreases in ET‐1 concentration were more pronounced by every dive.

## 4 DISCUSSION

This study has shown that repeatedly preformed rSCUBA diving (five dives, one per week, depth 20–30 m, total time 30 min, after a non‐dive period of 5 months) triggered an adaptive response of CV system and muscles, and most probably an activation of the immune response. The cumulative effect of five dives was reflected in statistically significant changes in the plasma or serum concentrations of cardiac damage (hs‐TnI, NT‐proBNP, and hs‐CRP), muscle damage (Mb and Gal‐3), vascular endothelial activation (ET‐1 and VEGF), and inflammatory (IL‐6) biomarkers. Furthermore, every single dive triggered statistically significant changes in the concentration of all these biomarkers, and also in the concentration of CK and LDH. The cumulative effect was not observed for Lkc, CRP, and CK‐MB, although a slight increase after the third dive was noted.

As it will be discussed further later, we assume that most of these changes occurred due to the adaptation of the organism to the aquatic environment and physical exertion, not hemorheological modification triggered by immersion and/or hyperbaric conditions. Namely, our recent study (Perović et al., 2020), on the same samples, revealed changes in some hemorheological parameters, for example, hemoconcentration that can influence certain parameters measured in this study. However, we observed decreases in some biomarkers, both after the dives and throughout whole monitored period, so we argue that the effects of hemorheological changes are general, and could only slightly increase all values, but which remain within the reference ranges.

Numerous studies have shown that exercise may cause a strong inflammatory response characterized by the mobilization of leukocytes, and an increase in circulating inflammatory mediators produced by immune cells and active muscle tissue (Cerqueira et al., 2020). However, stable levels of Lkc and CRP, in addition to unchanging pre‐dive IL‐6 levels, suggest that rSCUBA diving does not trigger systemic inflammation if practiced regularly, once a week for 1 month.

Nevertheless, a mild, but statistically significant increase in the IL‐6 concentration observed after every dive suggests activation of the immune response. Interestingly, the release of pro‐inflammatory cytokines (TNF‐α, IL‐1β, and IL‐6) after physical activity of sufficient intensity is followed by the release of anti‐inflammatory or regulatory cytokines (IL‐4, IL‐10, IL‐1 receptor antagonist, and IL‐13) that attenuate the response (Moldoveanu et al., 2001). Furthermore, in response to prolonged exercise, IL‐6 is synthesized by contracting the skeletal muscle in order to maintain energy status during exercise by stimulating glucose production and activating lipid metabolism through the stimulation of fatty acid oxidation (Hennigar et al., 2017). Thus, the increased energy needs of muscles as a part of adaptive response to the repeated diving could be a cause of the increment in IL‐6 increase observed after every dive.

rSCUBA diving is considered as a moderate‐intensity physical activity, and statistically significant increases in the concentration of Mb, CK, and LDH observed after every dive confirm myocyte activation followed by the disturbance of myocyte membrane integrity. Other studies have also found an increase in CK and LDH concentrations after SCUBA diving (Bilopavlovic et al., 2013; Ferrer et al., 2007). However, increases in the concentration of any of these parameters have not been pronounced as in high‐intensity physical exercise, for example, marathon running, in which values of these biomarkers multiply (Bernat‐Adell et al., 2019), suggesting the significantly lower extent of the disturbance of myocyte membrane integrity during diving. Physical activity triggers Mb release into circulation that is a consequence of protein degradation in the muscles (Cockburn et al., 2008). Mb concentration was found to be increased after the exercise (Ascensao et al., 2008) and diving (Žarak et al., 2020) within 30 min, most likely due to the peripheral vasoconstriction, membrane permeability impairment, and mild inflammatory process in the organ itself (Neubauer et al., 2008). Mb concentration correlates with a neutrophil response caused by stress on muscle tissue (Suzuki et al., 1999). Therefore, Mb can be considered as a useful biomarker for monitoring effectiveness of muscle work and adaptation to physical activity (Speranza et al., 2007). In our recent study (Žarak et al., 2020), we showed that SCUBA diving is followed by an increase in Mb concentration immediately after the dive, but a return to the basal level during the 6 hours recovery period was observed. Interestingly, in this study, the Mb concentration increments recorded after every dive reduced with time, suggesting that myocyte burden diminished, and muscles adapted to the physical activity.

Similar dynamics were obtained for the Gal‐3 values; even though its concentration was significantly higher after diving, the increments were smaller after every performed dive. Gal‐3, a β‐galactoside lectin with numerous functions (Dumic et al., 2006), is well known as a novel prognostic biomarker of heart failure (HF) with high predictive value for CV mortality and re‐hospitalization in HF patients (McCullough, 2014), whereas its low plasma concentration is associated with “successful” aging (Sanchis‐Gomar et al., 2016). Increased Gal‐3 values, similar in extent to those in different morbidities (Mueller et al., 2015), were also found after marathon (Hättasch et al., 2014) and ultramarathon running (Salvagno et al., 2014), but not after half marathon running (Vassalle et al., 2018). However, a study on mice suggested that the main origin of released Gal‐3 after physical activity is skeletal muscle, not cardiomyocytes (Hättasch et al., 2014). In this study, the Gal‐3 concentration remained below URL and followed the dynamics of Mb release that happens most probably because of changes in the permeability of the skeletal muscle membrane due to physical activity. Interestingly, when compared both pre‐ and post‐dive values, Gal‐3 concentration, like Mb concentration, decreased significantly after every performed dive, thus indicating skeletal muscle adaptation to continuously performed rSCUBA diving.

NT‐proBNP is a valuable biomarker of HF and indicator of stress and stretch within the myocardial walls caused by blood pressure overload (Scharhag et al., 2008), the events that can be also associated with SCUBA diving due to the immersion and high pressure of the environment. Yet, its active form, the hormone brain natriuretic peptide (BNP) has important physiological natriuretic, vasodilating, and sympatho‐inhibitory effects and can reduce the myocardial wall stress, *via* reducing heart preload and afterload. In accordance with our previous results (Žarak et al., 2020), in this study, we also found a statistically significant NT‐proBNP increase immediately after diving at every time point, partially due to hemodynamic (plasma volume change, fluid imbalance, and increase in central blood volume) and cardiac (increase of central venous pressure, stroke volume, and cardiac output) changes that occur during diving. Furthermore, these increments increased after every dive, which is in contrast to two other studies on divers practicing technical diving every day (Ljubkovic et al., 2010; Marinovic et al., 2010), in which a cumulative effect was not observed. These findings suggest that diving conditions (technical or recreational, depth, duration, breathing gas, *etc*.) and frequency, along with diver's characteristics (e.g., professional or recreational, experienced or beginner) could have an important role in triggering as well as activation intensity of underlying molecular mechanisms that lead to an adaptive response of particular tissue or organ. To summarize, it is reasonable to conclude that continuously performed rSCUBA diving could lead to the continuous release of BNP in order to protect the heart from damage and, if extended, to the physiological remodeling as a consequence of repetitive cardiac loading, which helps athletes to withstand the physical overload. It is also interesting to observe that an increase in BNP concentration leads to vasodilatation and reducing cardiac hypertrophy (Scharhag et al., 2006) by inhibition of the sympathetic nerves (D'Souza & Baxter, 2003), thus resulting in the protection of cardiac myocytes as presented in studies on animal and cell models (Filippatos et al., 2001).

The substantial increase of hs‐TnI observed after every dive could be related to the interplay of various factors such as the impairment of the cardiomyocyte membrane integrity (Lippi & Banfi, 2010), actions of free radicals production due to hyperoxia (Perovic et al., 2014) as well as increased catecholamines release due to the sympathetic nervous system activation triggered by long exposure to the coldness (Anegg et al., 2002). On the other hand, a decrease in both pre‐dive and post‐dive values after every performed dive suggests cardiomyocyte adaptation, and reduction of transitory necrosis and oxidative stress.

While an increased CRP concentration reflects systemic inflammation, CRP measured by a high‐sensitivity method has been proposed as an independent predictor of CV disease development (He et al., 2010; Pearson et al., 2003). Many studies associated a decrease in the circulating hs‐CRP with increased physical activity and exercise (Hammonds et al., 2016). In this study, hs‐CRP remained in the reference range, but its concentration slightly increased after every dive, and when both only pre‐dive and only post‐dive values were compared, a continuous increase was observed. This could be related to the early phase of the local inflammatory response in the heart tissue, due either to altered ventricular function (He et al., 2010) or a beginning of heart remodeling. Therefore, it would be interesting to monitor these changes over an extended period of time, as well as after the diving period, to elucidate if they are also part of the adaptive response and just a transient episode that would begin to diminish with time or the cessation of diving practicing is prerequisite for leading to the return to the basal level of hs‐CRP.

Although known as the strongest vasoconstrictor in the human body, ET‐1 also has pleiotropic effects in the heart and modulates its function by activating signaling pathways that impinge upon hypertrophic, proliferative, and cell survival responses (Schorlemmer et al., 2008). The observed significant decrease in the ET‐1 level immediately after every preformed dive can be ascribed to maintaining an adequate vascular tone during diving as well as the maintenance of prolonged vasodilatation in the recovery period. After the initial decrease, the ET‐1 concentration increased during the recovery period of 6 hours, but pre‐dive values were not reached (Žarak et al., 2020). Interestingly, whereas the ET‐1 pre‐dive values increased with time, the post‐dive values remained unchanged, thus making these drops even more pronounced. An increase in the ET‐1 pre‐dive values probably additionally upregulates the production of nitric oxide (NO), which has cardioprotective effects (Tawa et al., 2011). An adequate ratio of ET‐1 to NO during diving contributes to a proper perfusion of the cardiac myocardium and the maintenance of vascular tone, which additionally helps to reduce oxidative stress and damage. In addition, as ET‐1 affects the contractile properties and growth of the cardiac myocyte (Olfert et al., 2010), its continuous increase caused by rSCUBA could also be associated with the heart's remodeling and growing due to repetitive cardiac loading.

VEGF has been demonstrated to be crucial in activity‐induced angiogenesis (Richardson et al., 1999) that occurs due to the peripheral hypoxia, which also occurs in SCUBA diving (Perovic et al., 2014). During exercise and diving, the endothelium is constantly exposed to hemodynamic forces that vary in magnitude and direction and are dependent on the anatomical characteristics of the blood vessel. The action of these forces is considered as an important activator of endothelium, which results in remodeling but also the formation of new blood vessels (neovascularization) primarily under the influence of VEGF (Resnick et al., 2003). VEGF directly affects changes in the composition of the extracellular matrix and endothelial cell proliferation (Richardson et al., 2020). During exercise, neovascularization is most needed and expressed in skeletal muscle, in which VEGF exerts its angiogenic effects (Brodal et al., 1977; Hang et al., 1995). This allows muscle tissue to achieve better and faster oxygen transfer between the microcirculation and the mitochondria of the muscle itself. However, after initial increase of VEGF gene expression, prolonged exercise leads to the suppression of VEGF gene expression and decrease of VEGF that reflects the adaptation response of the organism to the physical activity (Gustafsson et al., 1999; Richardson et al., 2020). In conclusion, it seems that VEGF plays the most important role in the initial phase of the adaptation response to enhanced physical activity when pronounced angiogenesis occurs (Richardson et al., 2020). The same effect has been observed in our study, as the VEGF pre‐dive and post‐dive concentrations decreased after every performed dive. The adaptation mechanism is also supported by the fact that repetitive activity favors the selective expression of mRNA for VEGF receptor 2 (VEGFR2) (Gustafsson et al., 2007), which contributes to the angiogenic effect of VEGF (Neufeld et al., 1999).

### 4.1 Study limitations

To the best of our knowledge, this is the first study on the cumulative effect of rSCUBA diving on the blood concentration of the majority of the selected biomarkers, so some of the results could not be compared with the previously published data, or could be only compared with the data obtained for the divers practicing technical SCUBA diving, which have also been very scarce. Although the sample size was suitable for the high‐quality statistical analyses, which provided answers to all questions addressed in the study, it did not enable grouping of the divers by either age or diving experience per year for further analyses of these factors’ effects on the adaptive response to the repeated rSCUBA diving. The study did not include female subjects, thus the study of possible effects of sex on the adaptive response to the repeated rSCUBA diving have not been feasible. Further, this study was conducted over a relatively short period of 1 month; therefore, the study conducted under the same diving conditions over a longer period of time could reveal additional information on the cumulative effect of rSCUBA diving on the blood concentration of the selected biomarkers.

## 5 CONCLUSIONS

Under challenging environmental conditions, stress forces organisms to adapt to the rapidly changing surroundings. In this study, we showed that repeatedly performed rSCUBA diving (five dives, one per week) triggered activation of an adaptive response of the CV, muscular, and immune systems that were reflected in changes in the specific biomarkers. No clinically relevant impairment of any observed systems was seen, but all measured biomarkers showed different dynamics of change with repeatedly performed rSCUBA. Therefore, it would be interesting to monitor dynamics of changes in these biomarkers with rSCUBA diving performed repeatedly over a longer period of time, thus providing an opportunity either to confirm the observed adaptive response or to disclose possible adverse effects. Yet, studies should be carefully designed, because the frequency of repeated dives seems to be crucial for triggering the adaptive response that could be recorded by the blood levels of the specific biomarkers.

## CONFLICT OF INTEREST

The authors declare that they have no conflict of interest.

## AUTHOR CONTRIBUTION

MŽ, AP, and JD conceived and designed research. AP and MNJB conducted sample collection. MŽ, AP, and MNJB preformed sample analyses. MŽ and SŠG preformed statistical analysis. MŽ and JD wrote the manuscript. All authors read and approved the final version of the manuscript.
